# Bowel incarceration within the vaginal tunic in a three-and-half-year-old bilaterally cryptorchid Lhasa Apso

**DOI:** 10.1186/s13028-021-00586-y

**Published:** 2021-05-19

**Authors:** Dorcas Oyueley Kodie, Noah Segun Oyetayo, Oladotun Solomon Awoyemi, Cecelia Omowunmi Oguntoye, Oghenemega David Eyarefe

**Affiliations:** Department of Veterinary Surgery and Radiology, Faculty of Veterinary Medicine, University of Ibadan, Ibadan, Oyo State Nigeria

**Keywords:** Bowel obstructive emergency, Cryptorchidectomy, Tunica vaginalis

## Abstract

**Background:**

Cryptorchidism in dogs is of clinical concern due to its association with development of Sertoli cell tumours, seminomas and spermatic cord torsion. A patent inguinal ring has been found as a risk factor for peritoneal content migration and inguinal hernias. This study reports a case of bowel migration through a patent inguinal ring in a bilaterally cryptorchid dog and incarceration within the vaginal tunic of the left testicle.

**Case presentation:**

A three-and-a-half-year-old bilaterally cryptorchid Lhasa Apso with a history of anorexia, vomiting, stranguria and inability to defecate was diagnosed with bowel incarceration in the vaginal tunic of a retained left testicle. Surgery performed under epidural anaesthesia with acepromazine/butorphanol premedication revealed a loop of the colon entrapped in the vaginal tunic of the retained left testicle. The incarcerated bowel was thoroughly examined for viability and repositioned into the abdominal cavity. The inguinal ring was repaired and bilateral cryptorchidectomy performed.

**Conclusion:**

Cryptorchidectomy in dogs is often considered when there is concern for neoplasm or torsion of retained testes. However, this report suggests that cryptorchidectomy should be considered also to preclude the possibility of bowel obstructive emergencies.

**Supplementary Information:**

The online version contains supplementary material available at 10.1186/s13028-021-00586-y.

## Background

Cryptorchidism in dogs, a congenital failure of one or both testes to descend into the scrotum within 6 months after birth [1–3], is associated with a potential non-closure of the inguinal ring [2, 4]. The condition is of clinical concern as such dogs have an increased risk of developing Sertoli cell tumours, seminomas, and spermatic cord torsion [5, 6]. In male dogs’ normal reproductive organ development, the testes migrate from the abdominal cavity through the inguinal ring and descend into the scrotum following contraction of the gubernaculum under the influence of androgens secreted by Leydig cells [5, 7]. The tunica vaginalis is a pouch of serous membrane derived from the vaginal process of the peritoneum. It envelops the testis, its ducts and spermatic cord and continues through the inguinal canal, lining the interior of the scrotum [8]. When the testicles fail to descend, they may be located either in the abdominal cavity or inguinal canal [3, 4]. Following descent of the testes, the inguinal ring contracts (seals up). However, in cryptorchid dogs, the inguinal ring subsists as a potential opening, which has been viewed as a risk factor for inguinal hernias [9]. This may be further exacerbated by the low testosterone levels causing muscle weakness in bilaterally cryptorchid dogs [10, 11].

Upadhye et al. [12] reported a case of inguinal hernia in a unilaterally cryptorchid dog with omental herniation and attachment to the retained testicle. Here, we report a case of bowel incarceration in the vaginal tunic in a bilaterally cryptorchid dog. To the author’s knowledge, this is the first case of bowel entrapment within the vaginal tunic associated with a patent inguinal ring of a cryptorchid dog. This presents the patent inguinal ring as a possible risk factor for bowel emergencies.

## Case presentation

A three-and- half-year-old bilaterally cryptorchid Lhasa Apso weighing 6.8 kg was presented to the Veterinary Teaching Hospital, University of Ibadan, Nigeria with a painful subcutaneous swelling at the left inguinal area. The owner’s complaints included vomiting for two days prior to presentation, anorexia, stranguria and inability to defecate.

**Fig. 1:**
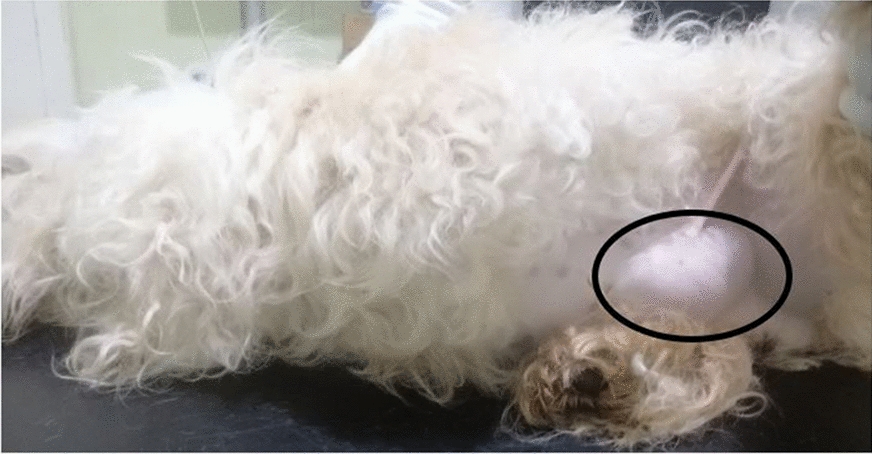
Image of patient showing swelling at the left inguinal area (indicated by circle)

**Fig. 2:**
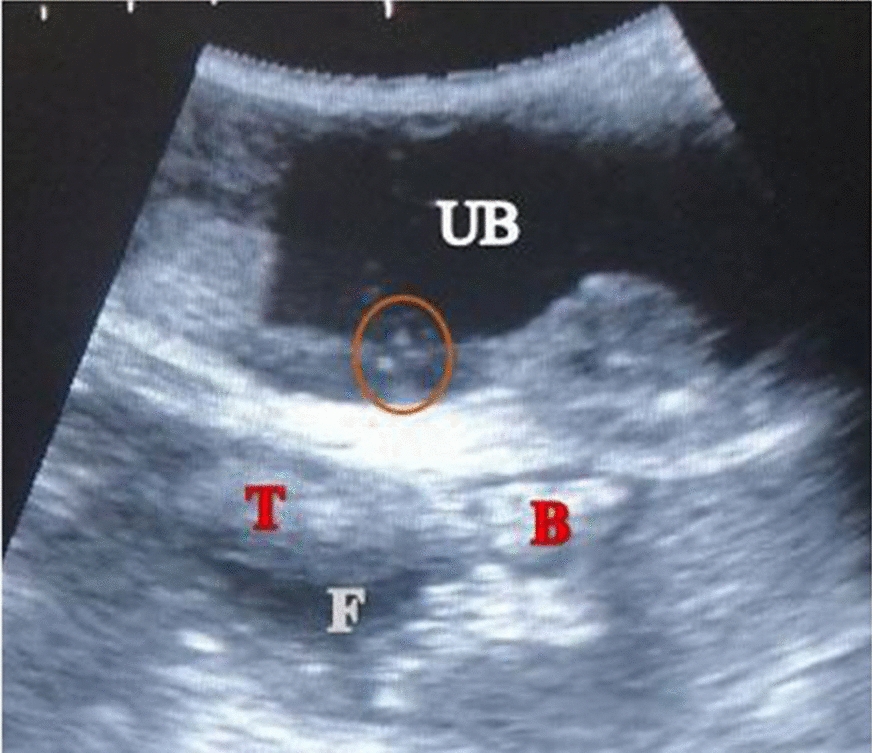
Sonogram of the inguinal region. A fluid-filled sac (indicated by anechoic area marked F) containing the left testicle (hypoechoic area marked T) and a segment of bowel within the vaginal tunic (hyperechoic area marked B). Few small-sized calculi were also observed (indicated by circle) in the urinary bladder (UB)

**Fig. 3:**
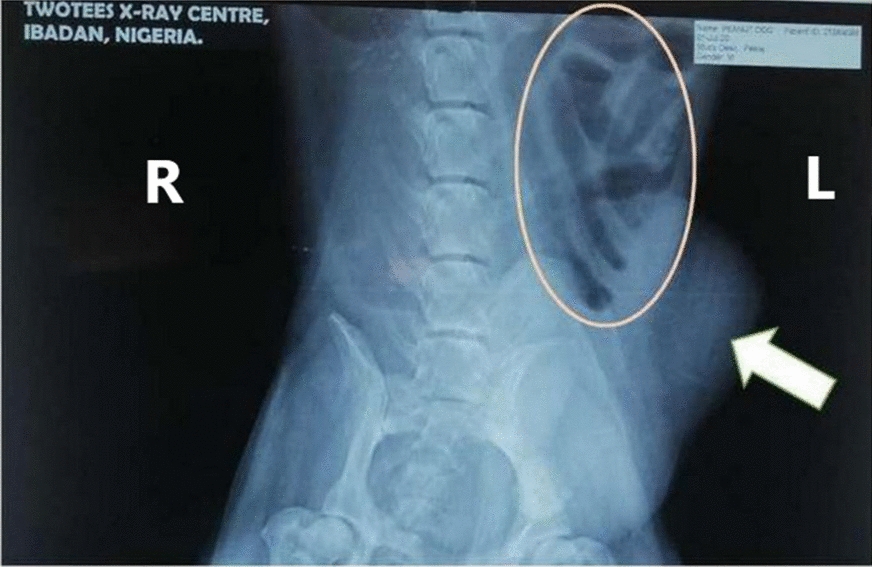
Dorsoventral radiographic view of the abdomen. Abdominal radiograph revealed area of fluid opacity (arrow) and gas-filled loops of intestine (circle) cranial to the area of swelling

At presentation, the patient was lethargic and inactive. Rectal temperature: 37.9 °C; heart and pulse rates: 92 beats per minute; normal capillary refill time (CRT < 2 s) and with a slightly higher than normal respiratory rate (112 breaths per minute). A large sausage-like swelling measuring 6.9 × 4.5 cm was observed at the left inguinal area, cranio-lateral to the prepuce (Fig. 1), which elicited pain response upon palpation. Haematology and biochemical analyses revealed mild band-neutrophilia, lymphopenia and hyperglobulinaemia, with slightly elevated blood urea nitrogen (BUN) and creatinine (azotaemia). All the other haematological and blood chemistry values were within normal reference ranges (Additional file 1). Urine collected via catherisation revealed haematuria, moderate proteinuria and mild bilirubinuria, and presence of phosphate crystals in the slightly acidic urine (pH 6) (Additional file 2). Ultrasound examination revealed a fluid-filled sac at the inguinal region enclosing the retained left testicle and a loop of bowel. Few small-sized calculi were also observed in the urinary bladder (Fig. 2). An abdominal radiograph revealed an area of fluid opacity at the left inguinal region and gas-filled loops of intestine cranial to the swelling (Fig. 3), indicative of an obstruction. The patient was prepared for emergency surgery.

**Fig. 4:**
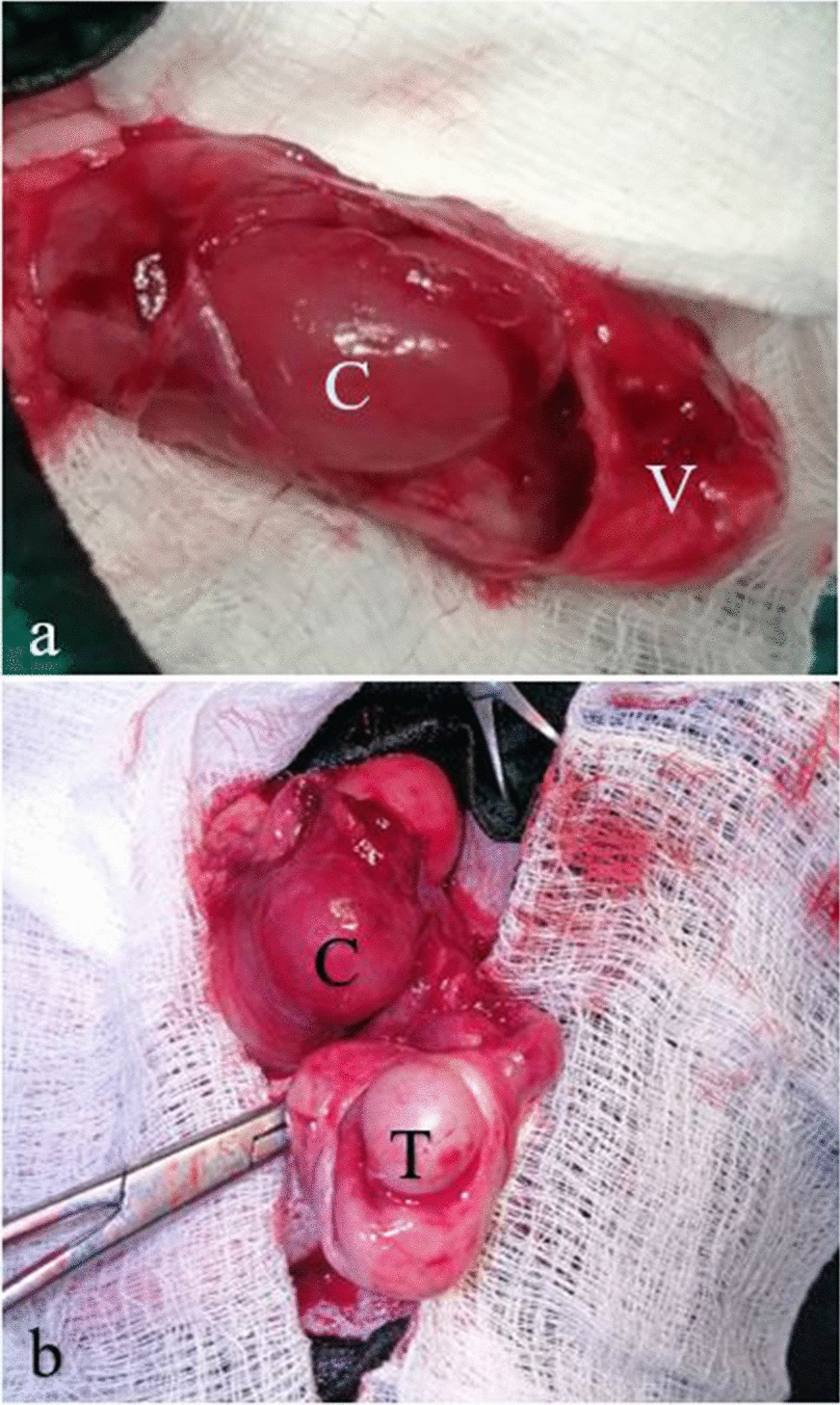
Intraoperative images of the incised swelling. **a** Shows the incarcerated loop of colon (C) observed within the vaginal tunic (V). **b** Shows the exposed retained left testicle (T) and the loop of colon (C), both observed within the vaginal tunic

**Fig. 5:**
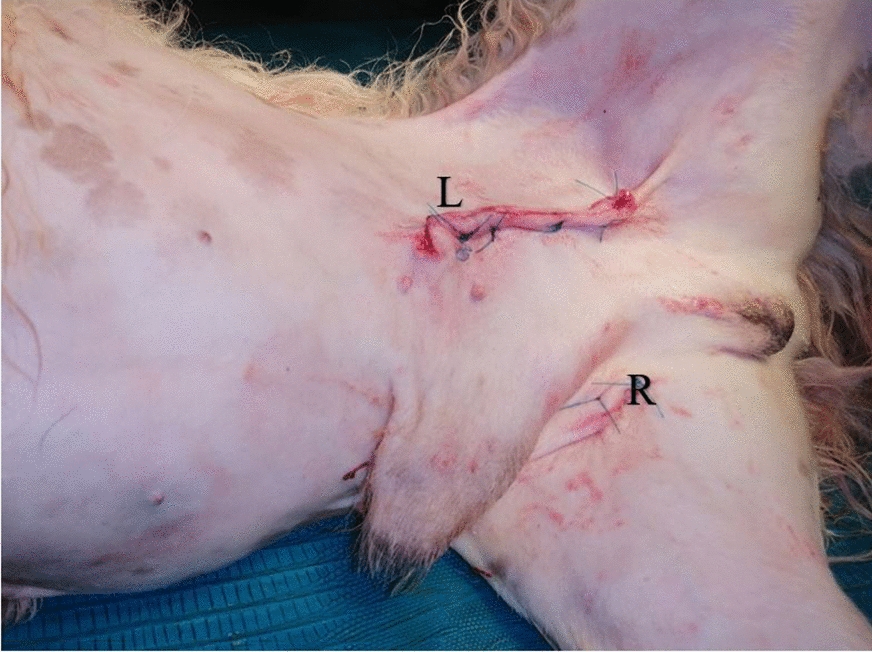
Image of patient after bilateral cryptorchidectomy. The positions of the testes prior to removal are indicated by R for the right testicle and L for the left

**Fig. 6:**
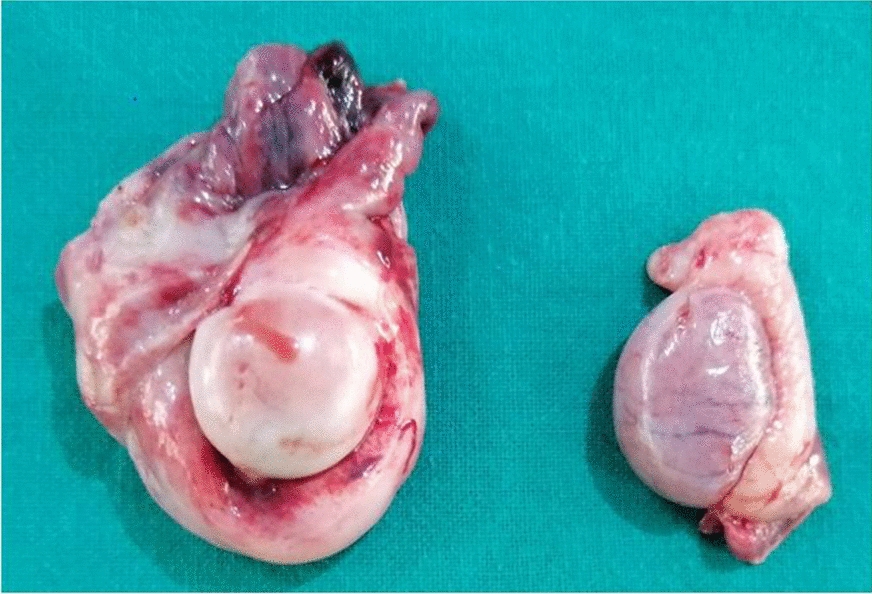
Image of the left and right testicles after cryptorchidectomy. The left testicle, as well as the vaginal tunic (on the left) appeared remarkably larger than the right due to thickening of the tunica vaginalis tissue

Patient peri-operative stabilisation process was achieved with lactated Ringer’s solution (Ashmina Ltd, Nigeria) and antibiotic therapy (amoxicillin, Pamoxil, Yanzhou Xier Kangtai Pharmaceutical Co. Ltd., China) at 10 mg/kg administered intravenously via the cephalic vein through a pre-placed gauge 21 scalp vein set. The patient was premedicated with 2% acepromazine (Novartis Animal Health UK Ltd, UK) and 1% butorphanol (Torbugesic, Fort Dodge, USA) administered via intramuscular injection, both at doses of 0.1 mg/kg. Epidural nerve block was achieved with 2% lidocaine (Andralocain + AD, Amakin Pharmaceutical Limited, India) and 0.5% bupivacaine (Duracaine, Myungmoon Pharm. Co. Ltd, China) (Ratio 1:1) as previously described [13]. The patient was aseptically prepared, placed in dorsal recumbency and draped for surgery. A 5 cm skin incision was made over the swelling at the left inguinal area, cranio-lateral to the prepuce. After incision of the swelling with a scalpel blade size 15 and opening of the swelling with a Metzenbaum scissor, 25 mL of fluid was aspirated. Upon exposure, the left testicle covered with tunica albuginea was observed at the distal end of the blind sac (vaginal tunic). Proximal to it and overlying the testicular vessels was a small loop of colon projecting into the proximal portion of the tunic about 2 cm from the outer inguinal ring and anchored in place by a small hard faecal pellet. The incarcerated colon portion which enclosed the faecal pellet was congested (Fig. 4). Adhered mesentery was detached, and the incarcerated bowel loop was removed from the vaginal tunic. The faecolith was moved caudally and the entrapped loop was examined for viability. An observed mesenteric tear was repaired with 2–0 polyglycolic acid (Covidien Ireland Limited, USA) in a simple continuous pattern. The bowel segment was copiously lavaged with warm normal saline solution and returned into the abdominal cavity. The retained left testicle as well as the vaginal tunic were ligated with size-0 chromic gut (Anhui Kangning Industrial Ltd, China), transected and submitted for histopathological examination. The inguinal ring was repaired with 2–0 polyglycolic acid (Covidien Ireland Limited, USA) using a simple interrupted suture pattern. The incision on the swelling was closed routinely in three layers with 2–0 polyglycolic acid (Covidien Ireland Limited, USA) for the muscles and subcutis, and size 2–0 nylon sutures (Huaiyin Medical Instruments Co. Ltd, China) for the skin as earlier described [4]. Cryptorchidectomy was performed for the right testicle as well, which was palpated caudolateral to the prepuce (Figs. 5, 6).

Following surgery, oral amoxicillin (Amoxil, Medreich Ltd, India) was prescribed to be administered for 5 days at a dosage of 15 mg/kg twice daily. The patient recovered uneventfully, and sutures were removed after 2 weeks. The client was also advised to feed a specialised diet (Royal Canin^®^ Urinary SO) to aid in dissolving the urinary calculi observed at ultrasonography.

The result of histopathological evaluation revealed accentuation of the tunica albuginea, marked testicular hypoplasia with the tubules showing Sertoli cell only pattern and complete lack of germ cells. Some of the tubules were also degenerated, distorted with thickened basement membrane, and hyalinised. There was moderate interstitial fibrosis, and accentuation of interstitial endocrine cells.

## Discussion and conclusion

Bowel incarceration is a surgical emergency [14] with clinical signs characteristic of acute abdomen [15], a term used to describe sudden onset of abdominal pain and discomfort [4, 16]. Clinical signs are non-specific and include depression, anorexia, abdominal pain, vomiting and/or diarrhoea [15, 16]. Identification of the specific cause of the condition in dogs is often unattainable without thorough laboratory and proper diagnostic imaging techniques [17]. In this case, the patient was presented with depression, anorexia, abdominal pain and vomiting, all of which are classic signs of acute abdomen. This necessitated the use of ultrasonographic and radiologic techniques which revealed bowel obstruction, evident on the radiograph by the gas-filled loops of intestine cranial to the area of swelling and on the sonogram by hypoechoic bowel segment bathed in anechoic fluid within a hyperechoic sac. The mild band-neutrophilia, lymphopenia and hyperglobulinaemia observed were classical bone marrow signalling responses to mitigate infection in such obstructive enterocolitis and enterotoxaemia caused by the incarcerated bowel [18].

The exact aetiology of the condition in this case was idiopathic. The patent inguinal ring in cryptorchid dogs has been reported as a predisposing factor in the development of inguinal hernias [3, 4]. Aside congenital body wall defects, trauma and increased intra-abdominal pressure have been documented [19]. The accidental diagnosis of urinary calculi with associated stranguria may be connected with aetiology of the condition in this patient. Lhasa Apsos are predisposed to struvite and calcium oxalate calculi [4], which may have resulted in the pre-renal azotaemia observed in this case. Calculi-induced stranguria with associated straining causes increased intraabdominal pressure and bowel hypermotility [20]. The bowel peristaltic force may have forced the segment of colon into an apparent patent inguinal ring with a likely defective vaginal process resulting in the entrapment of the colon segment within the vaginal tunic.

Cryptorchidism is a condition in dogs with multifactorial aetiologies including heredity. In spite of several studies to understand the genetic and molecular causes of the condition, the specific mechanisms leading to canine cryptorchidism are still unclear [9]. As a consequence, unilaterally cryptorchid dogs, though sometimes fertile, are discouraged from being bred to prevent passing on the implicated recessive gene trait to their offspring [21], and resultant development of Sertoli cell tumours or seminomas later in life [5, 6]. In this case, the left testicle appeared to be larger than the right (Fig. 6). The histological findings indicated testicular degeneration and hypoplasia characteristic of retained testicle without evidence of testicular tumour. The apparently larger size of the left testicle was therefore due to thickening and congestion of the tunica albuginea.

The development of inguinal hernias in cryptorchid dogs has been well documented [2–7, 9]. However, cases involving both testes and abdominal content enclosed in the hernia sac are rare. Upadhye et al. [12], in their report, observed omental herniation and attachment to a retained testicle in a unilaterally cryptorchid pug, but outside of the vaginal tunic. The peculiarity of this case is the herniation of a segment of the colon through the inguinal ring and subsequent incarceration within the vaginal tunic. The incarceration was also aided by the entrapment of the small pellet of faecolith, observed at surgery, which prevented the movement of the entrapped segment out of the vaginal tunic. This possibility of abdominal content dislocation through the patent inguinal ring of cryptorchid dogs and subsequent bowel incarceration poses a risk of bowel obstruction emergencies. It is therefore the authors’ recommendation that cryptorchidectomy be considered to also preclude the possibility of bowel obstructive emergencies in cryptorchid dogs.

## Supplementary Information

**Additional file 1:** The patient’s full haematological and clinical chemistry report.

**Additional file 2:** Patient’s urinalysis report.

## Data Availability

Data sharing is not applicable to this article as no datasets were generated or analysed during the present case.

## Declarations

### Prior publication

The present case has not been previously published.
