# Supplementary material for: Bowel incarceration within the vaginal tunic in a three-and-half-year-old bilaterally cryptorchid Lhasa Apso
Source: Acta Vet Scand. 2021 May 19;63:21. doi: 10.1186/s13028-021-00586-y (PMC8136335; doi:10.1186/s13028-021-00586-y)
Supplement: Supplementary file 1 — Additional file 1: The patient’s full haematological and clinical chemistry report. [file 13028_2021_586_MOESM1_ESM.docx]

| **Additional file 1**: The patient’s full haematological and clinical chemistry report | | |
| --- | --- | --- |
| Parameter | Value | Normal range |
| Haemoglobin (g/dL) | 16.8 | 12-18 |
| Packed cell volume (%) | 52 | 37-55 |
| Red blood cells (10^6^ /µL) | 8.33 | 5.5-8.5 |
| Mean corpuscular volume (fl) | 62 | 60-77 |
| Mean corpuscular haemoglobin concentration (g/dL) | 32 | 32-36 |
| Platelets (× 10^5^ /µL) | 2.01 | 2-9 |
| Total White Blood Cells (× 10^3^ /µL) | 9.05 | 6-7 |
| Segmental Neutrophils (%) | 76 | 60-70 |
| Band Neutrophils (%) | 6 | 0-3 |
| Lymphocytes (%) | 10% | 12-30 |
| Monocytes (%) | 7 | 3-10 |
| Eosinophils (%) | 0 | 2-20 |
| Basophils (%) | 0 | Rare |
| Total Protein (g/dL)  Albumin (g/dL)  Globulin (g/dL) | 7.3  3.2  4.1 | 5.4 – 7.5  2.3 – 3.1  2.7 – 4.4 |
| Alanine Aminotransferase (u/L) | 109 | 10 – 109l |
| Aspartate transaminase (u/L) | 20 | 13 – 15 |
| Alkaline Phosphatase (u/L) | 117 | 1 – 114 |
| Creatinine (mg/dL) | 1.9 | 0.5 - 1.7 |
| Blood urea nitrogen (mg/dL) | 34 | 8 – 28 |
