# Supplementary material for: Bowel incarceration within the vaginal tunic in a three-and-half-year-old bilaterally cryptorchid Lhasa Apso
Source: Acta Vet Scand. 2021 May 19;63:21. doi: 10.1186/s13028-021-00586-y (PMC8136335; doi:10.1186/s13028-021-00586-y)
Supplement: Supplementary file 2 — Additional file 2: Patient’s urinalysis report. [file 13028_2021_586_MOESM2_ESM.docx]

| **Additional file 2**: Patient’s urinalysis report | | |
| --- | --- | --- |
| Parameter | Value | Normal range |
| **PHYSICAL** |  |  |
| Colour | Yellow |  |
| Turbidity | Clear |  |
| Specific gravity | 1.008 | 1.016 – 1.060 |
| **DIPSTICK** |  |  |
| pH | 6 | 5.5-8.5 |
| Blood | + | - |
| Protein | 2+ | - |
| Bilirubin | + | - |
| Glucose | - | - |
| Ketones | - | - |
| **SEDIMENTS** |  |  |
| RBC (HPF) | 3-5 HPF |  |
| WBC (HPF) | 2-10 HPF | 0-5 HPF |
| Casts (Types) | 1-3 HPF (Hyaline) |  |
| Crystals | 1-2 HPF(Phosphate) |  |
| Bacteria | - |  |

(HPF = high power field)
